# Melatonin as a Therapy for Traumatic Brain Injury: A Review of Published Evidence

**DOI:** 10.3390/ijms19051539

**Published:** 2018-05-22

**Authors:** Nicole Osier, Emily McGreevy, Lan Pham, Ava Puccio, Dianxu Ren, Yvette P. Conley, Sheila Alexander, C. Edward Dixon

**Affiliations:** 1 School of Nursing, University of Texas at Austin, Holistic Adult Health, Austin, TX 78712, USA; emily@mcgreevy-family.com; 2 Department of Neurology, Dell Medical School, University of Texas at Austin, Austin, TX 78705, USA; 3 School of Nursing, University of Pittsburgh, Pittsburgh, PA 15213, USA; lhp4@pitt.edu (L.P.); PuccAM@upmc.edu (A.P.); dir8@pitt.edu (D.R.); yconley@pitt.edu (Y.P.C.); salexand@pitt.edu (S.A.); 4 Department of Neurological Surgery, University of Pittsburgh Medical Center, Pittsburgh, PA 15260, USA; 5 Department of Human Genetics, University of Pittsburgh, Pittsburgh, PA 15213, USA; 6 School of Medicine, University of Pittsburgh, Pittsburgh, PA 15261, USA; 7 Safar Center for Resuscitation Research, University of Pittsburgh, Pittsburgh, PA 15213, USA; dixoec@upmc.edu; 8 V.A. Pittsburgh Healthcare System, Pittsburgh, PA 15240, USA

**Keywords:** melatonin, therapy, traumatic brain injury (TBI), neurotrauma, preclinical, review

## Abstract

Melatonin (MEL) is a hormone that is produced in the brain and is known to bind to MEL-specific receptors on neuronal membranes in several brain regions. MEL’s documented neuroprotective properties, low toxicity, and ability to cross the blood-brain-barrier have led to its evaluation for patients with traumatic brain injury (TBI), a condition for which there are currently no Food and Drug Administration (FDA)-approved therapies. The purpose of this manuscript is to summarize the evidence surrounding the use of melatonin after TBI, as well as identify existing gaps and future directions. To address this aim, a search of the literature was conducted using Pubmed, Google Scholar, and the Cochrane Database. In total, 239 unique articles were screened, and the 22 preclinical studies that met the a priori inclusion/exclusion criteria were summarized, including the study aims, sample (size, groups, species, strain, sex, age/weight), TBI model, therapeutic details (preparation, dose, route, duration), key findings, and conclusions. The evidence from these 22 studies was analyzed to draw comparisons across studies, identify remaining gaps, and suggest future directions. Taken together, the published evidence suggests that MEL has neuroprotective properties via a number of mechanisms with few toxic effects reported. Notably, available evidence is largely based on data from adult male rats and, to a lesser extent, mice. Few studies collected data beyond a few days of the initial injury, necessitating additional longer-term studies. Other future directions include diversification of samples to include female animals, pediatric and geriatric animals, and transgenic strains.

## 1. Introduction

Traumatic brain injury (TBI) affects countless individuals worldwide [1,2,3,4,5,6,7,8,9], with an estimated 2.5 million cases in the United States during 2010 alone [1]. Available treatments are not appreciably relieving symptoms and the quest for new therapies has not led to a single Food and Drug Administration (FDA)-approved therapy and, consequently, TBI patients often suffer long-term disabilities. Therefore, identifying novel TBI therapeutics that are safe and effective remains crucial. 

One promising candidate is melatonin (MEL), a naturally occurring hormone that is also available as an over-the-counter dietary supplement. MEL possesses many of the fundamental characteristics of a central nervous system (CNS) therapeutic including low toxicity [10,11,12], an ability to cross the blood-brain-barrier [13,14,15], and receptors that bind MEL in the CNS [16,17,18,19]. While the therapeutic effects of MEL in human TBI remain understudied, MEL has demonstrated beneficial effects in pre-clinical models of several CNS disorders, including conditions with similar pathology and symptoms profiles, to TBI such as: Huntington’s disease [20], Alzheimer’s disease [21], amyotrophic lateral sclerosis [22,23], stroke [24,25], sepsis-induced brain dysfunction [21], and spinal cord injury [26,27]. In the aforementioned contexts, MEL reduces secondary injury (e.g., apoptosis, inflammation, and oxidative stress) as well as reduces symptoms and functional deficits (e.g., memory, learning, and motor) [21,26,27,28]. It is possible that in the context of TBI, MEL will have similar improvements in secondary injury pathways and reduce symptom burden, which may contribute to other beneficial outcomes such as earlier return to play for sports-related injuries. 

There remains a significant gap in the literature pertaining to the role of therapeutic MEL after TBI due to direct injury to the brain or skull. Moreover, no recent review has comprehensively evaluated the TBI knowledge base as it pertains to MEL. The most comprehensive review available on this topic was published by Samantaray and colleagues in 2009, prior to publication of 13 of the articles included in this review. A more recent review of melatonin as a therapy for central nervous system injury was published by Naseem & Parvez (2014), but this review emphasizes spinal cord injury and cites only 4 TBI studies. The purpose of this review is to provide a comprehensive review which highlights the state-of-the-science as it pertains to the published evidence of pharmaceutical applications of MEL as a TBI therapy. Published evidence is summarized in tabular form, including sample (sample size, species, and characteristics), groups (number of groups, number of subjects per group; treatment of groups), results (outcomes and details regarding how and when they were assessed), conclusions, and limitations. This review will also identify gaps in the available evidence and suggest future directions for ongoing research. 

## 2. Results

In total, more than 200 unique articles were identified using the key word searches, alone or in combination (*n* = 239). Of these, articles were further screened using the following exclusion criteria: (1) written in a language other than English; (2) tested a condition beyond traumatic injury directly to the brain or skull (e.g., stroke; sepsis-induced neurological deficits; chemically-induced injury); (3) did not test the effect of melatonin; or (4) did not use an in vivo model (e.g., neuronal stretch model or another in vitro model). After applying the aforementioned exclusion criteria, a total of 22 articles remained (See Table A1, Table A2, Table A3, Table A4, Table A5, Table A6, Table A7, Table A8, Table A9, Table A10, Table A11, Table A12, Table A13, Table A14, Table A15, Table A16, Table A17, Table A18, Table A19, Table A20, Table A21 and Table A22). The studies analyzed a variety of TBI conditions, ranging from mild (Table A7 and Table A21) to moderate (Table A3 and Table A17). The remainder of the studies did not specify the severity of the condition. 

The global interest in evaluating the therapeutic effects of MEL after TBI is evident by the worldwide research conducted on this topic, including 8 studies from Turkey [20,24,29,30,31,32,33,34], 4 from the United States [35,36,37], 3 from China [38,39], 2 from Iran [40,41], 2 from Israel [42,43], 1 from France [44], 1 from Germany [44], and 1 from Italy [45].

The majority of the studies used rats, most commonly Sprague-Dawley rats [29,30,31,35,36,39,46,47,48] or Wistar rats [20,24,32,33]; *N*-Mary rats were used in 1 study [41] and NMRI rats used in another [40]. Less commonly, mice were used as the test animals, including the following strains: CD1 mice [39,45], Sabra mice [42], BALB/c [34], C57BL/6 mice [49,50], and Swiss mice [44]. Most of the studies conducted experiments on adult test animals with two exceptions testing the effects of childhood TBI [20,24]; one study did not specify the age of the test animals [33]. None of the studies explicitly included female mice in their sample, though some studies did not report the sex of the test animals [20,24,33,50].

Several different injury models were used to garner evidence regarding the therapeutic effects of MEL after TBI. Most commonly used was weight drop injury, which was employed in 12 of the studies [20,24,29,30,31,32,33,38,41,42,43,44]. Of the studies using weight drop, several methods and modifications were used including: Marmarou’s model, Feeney’s model, and Marklund’s model. The second most common method of inducing TBI in the MEL literature was controlled cortical impact (CCI), which was used in 6 studies [36,39,44,45,50,51]. Less commonly used models include surgical brain injury [35], fluid percussion injury [46], and cold-probe injury [34].

Most of the studies tested the effect of melatonin alone. Some of the studies tested multiple doses of MEL or several dosing schedules [20,24,33,35,39,40,41,48]. A small number of studies compared the effects of MEL to another drug with known neuroprotective (e.g., anti-oxidant) properties [20,42,43]. Five studies tested the effects of MEL in combination with another drug [34,38,45,46]. This evaluation of combination therapy has been suggested as a direction for future TBI researchers, since TBI pathophysiology is complex and may not be realistically targeted with a single drug [52]. 

Most of the studies tested the effects of MEL on cellular and histopathological outcomes, including but are not limited to: edema, markers of oxidative stress, inflammatory cytokines, lesion size, and mitophagy. Half of the studies tested the effect of MEL on one or more behavioral or functional outcomes after TBI [20,24,30,35,36,42,45]. The behavioral outcomes assessed in the studies included cognitive function assessed using the Morris water maze and motor function assessed using the Beam balance test, wire grip test, or a composite neuroscore.

While the therapeutic effects of MEL in human TBI remain understudied, some characterization of endogenous MEL after TBI has been published. One study [53] collected cerebrospinal fluid (CSF) and serum out to 13 days post-TBI and compared melatonin levels to that in controls who had a CSF sample collected during surgery for a condition distinct from TBI. CSF MEL fluctuated over time in a biphasic fashion, gradually increasing until day 2 post-TBI and then progressively decreasing to a minimum on day 5 with levels reaching a maximum on day 8. Overall, and for all days except of 0, 1, and 4, CSF MEL levels were significantly higher in TBI patients than controls. Serum MEL levels also increased from admission to day 2 post-TBI and then reached a minimum level on day 5; no significant differences in serum MEL levels between TBI patients and controls were reported [53]. A second study [54] examined MEL levels in the blood 8 times per day for the first two days after TBI but lacked a control group. MEL concentrations in the blood were lower than established clinical ranges and diurnal variation in MEL levels were associated with neurological outcomes assessed using the Glasgow Coma Scale (GCS); specifically, patients with lower GCS showed disrupted diurnal variation in MEL levels compared to those with higher GCS [54]. A third study [30] recruited TBI survivors 6 months after the initial diagnosis and compared their salivary MEL levels to non-TBI controls. Samples were collected under tight experimental controls including dim light (<10 lux), posture, and activity. Every half hour between 6:00 pm and 12:30 am participants provided saliva samples which were frozen at −20 °C until further analysis. When 14 TBI survivors and 14 controls were compared, there was no significant difference in dim light melatonin onset; however, when the total melatonin production over the entire sampling period was compared, significantly higher melatonin production (*p* = 0.031) occurred in controls [16]. Overall, the evidence suggests MEL levels go up then down in the acute period and may remain depressed chronically. Taken together, it is plausible that MEL is involved in the body’s response to TBI but may be inadequate for post-TBI neuroprotection [16,53,54].

### Clinical Studies

While additional pre-clinical research is needed, effort to translate MEL to clinical trials has begun. Specifically, there is an ongoing clinical trial in Canada exploring the effects of therapeutic MEL after pediatric TBI [55]. The pilot work underlying this clinical trial used a retrospective chart review of 48 pediatric TBI patients being treated for post-traumatic headache. The authors found that 18 (37.5%) of the patients received MEL therapy, suggesting this drug is administered after TBI in some units, despite the lack of clinical trials and limited pre-clinical evidence. Of the 18 patients receiving MEL, 15 (83%) demonstrated a therapeutic response. Since this was a retrospective study, there was no control over therapeutic regimen: 7 patients received doses between 3–5 mg and 8 patients received a dose between 6–10 mg. The ongoing clinical trial entitled the Post-concussion Syndrome in Youth—Assessing the gamma-aminobutyric acid (GABA)-ergic Effects of Melatonin (PLAYGAME) study, which will build on these pilot findings using a randomized control trial. As of 18 April 2018, this study is still active, but has stopped recruiting; it was previously enrolling males and females, aged 8–19, with mild TBI. The study will test the effects of either 3 mg or 10 mg of MEL administered sublingually for 28 days after trauma on symptoms of post-concussion syndrome assessed using the Post-Concussion Inventory (PSCI) score for parents (PSCI-P) and youth (PSCI-Y), the child health questionnaire for both parents and children, the behavior assessment system, and the behavior rating inventory of executive function. In addition to the effects of each dose on outcomes, the authors will explore a possible dose-response effect, as well as whether the effect of treatment is independent of the effect of sleep. The proposed PLAYGAME TRIAL end date is November of 2019.

A second clinical trial [52] recruited 7 male TBI patients age 16–65 (average = 39.6 years) who had previously sustained a TBI and suffered a post-TBI history of sleep disturbance. All of the participants were fully oriented and none had a history of neurological insults or dependence on drugs and/or alcohol. Potential test subjects were excluded if they were taking amitriptyline, a drug administered during the study. In this study, a randomized double-blind controlled cross-over trial was used to compare the effects of two drugs: 5 mg of MEL or 25 mg of amitriptyline. In this study, the effects of drug therapy were modest; MEL improved daytime alertness but did not improve sleep latency, sleep duration, or sleep quality, nor did it improve mood-related measures or cognitive performance. Response bias is an issue in this study as self-reports were not entirely consistent with the diaries used in data collection. Additional studies testing the effects of MEL after TBI are needed.

## 3. Discussion

### 3.1. Summary

Taken together the pre-clinical evidence surrounding MEL remains conflicted. The majority of studies showed some beneficial effect of MEL using one or more dosing regimens; however, one study [38] found no beneficial effect of MEL. Moreover, some studies showed dose-response effects. Commonly, a bell-shaped curve was reported with the middle dose, conferring benefit, and low and high doses ineffective [43,44]. Notably, the effective doses differed, with one study finding 1.25 mg/kg of MEL (but not 0.625 mg/kg or 2.5 mg/kg) effective [44], and another study finding 5 mg/kg (not 1 mg/kg or 10 mg/kg) effective [43]. Moreover, some of the regimens studied actually compounded the deleterious effects of injury. In one study, a dose of 150 mg/kg of MEL worsened edema, lipid peroxidation, vibrissae stimulation study, beam balance performance, and composite neuroscore [35]. In a second study, a dose of 200 mg/kg of MEL increased lipid peroxidation [32]. Other studies tested more than one dose of MEL but did not report dose-dependent effects [40,42]. Further research is necessary to understanding these adverse effects, especially since doses that led to adverse effects in one study were associated with neuroprotection in other studies [32,44,46]. Moreover, five of the studies [34,39,45,46] tested the effects of MEL in combination with another drug and found the effects of combined therapy were superior to the effects of either drug alone; this is consistent with a growing body of evidence that two or more drugs in combination may be needed to adequately treat TBI [52]. All in all, the majority of the authors’ research supports the conclusion that the use of MEL as a method for treatment of TBI, if used under appropriate conditions at an appropriate dosage, can be beneficial. Taken together, the evidence is limited and requires replication and expansion of published findings.

### 3.2. Critical Analysis of Published Studies

Overall, the pre-clinical knowledge base is limited by the lack of a well-tested conceptual framework surrounding the neuroprotective mechanisms of MEL; this limitation affects the researcher’s ability to design studies and contributes to the inconsistent results in published studies. Additional research testing MEL as a potential therapeutic agent is necessary to support clinical trials and translational efforts. Traditionally, the beneficial effects of MEL within the CNS are attributed to its anti-oxidant capacity [10,11,12,13,14,56]. Anti-apoptotic effects of MEL have also been reported in the CNS [4,9,21,25,57], including after TBI [9,20,21,22,23,24,25,35,36,38,43,44,45]. One study found MEL therapy promoted mitophagy after TBI [47].

It is also worth noting that none of the studies reviewed acknowledged the role of endogenous MEL receptors, including MT1 and MT2, two MEL-specific receptors found in the mammalian brain [37,38]. This represents a limitation of the research since MEL receptor levels have been found to be reduced after TBI [57] and evidence from a mouse model of Huntington’s disease suggests the anti-apoptotic effects of MEL are MT1 receptor-dependent [21]. The role of MT1 and MT2 remains unclear in the context of TBI and may be relevant to personalized medicine since functional polymorphisms in MT1 have been identified [26,56].

### 3.3. Limitations and Methodological Inconsistencies of Published Studies

Examination of the existing body of literature has led to identification of a major limitation in TBI research; that being that most studies are limited to the acute effects of TBI assessed only within a few days of initial injury. Only four studies [3,20,24,32,47] examined outcomes at or beyond 2 weeks post-injury. Notably, the longest data-collection period of the studies included in this review was 3 weeks post-TBI [20,24]. Future studies will be strengthened by outcome assessment into the chronic period including studies collecting data beyond 3 weeks of the initial injury.

Another limitation in the existing evidence surrounds the type of test animals used. A lack of inclusion of female test animals represents a limitation of the existing knowledge base and an area for future research. While males are more likely to sustain a TBI in all age groups [9,58], female TBI remains a significant worldwide problem stemming from domestic violence as well as traditional injury mechanisms (e.g., motor vehicle accidents; falls). Still, this overrepresentation of males in experimental TBI studies represents a limitation of the evidence; notably, to address this common limitation, many research funding agencies are now requiring that studies include female test animals or sufficiently justify exclusion from the sample. 

Similarly, there was an overrepresentation of adult test animals in the studies reviewed with only two studies [20,24] focusing specifically on juvenile animals and none focusing on aged or senescence-prone animals. Additional research using representative pre-clinical samples is necessary to promote high-quality evidence that is translatable to clinical trials and ultimately applicable to the TBI patient population. Regardless of the test animals used, it is critical that details pertaining to sex, age, strain, and weight be reported as consistent with the Common Data Elements (CDEs) published by the National Institute of Neurological Diseases and Stroke (NINDS) [59]. Not all of the studies reviewed adequately provided these details that would be necessary to replicate the research. Another detail that was occasionally omitted from the studies in this literature review was diet [33,44,47,48], though most studies specified that animals had *ad libitum* access to food and water.

An important consideration for studies examining the melatonergic system or testing how therapeutic MEL pertains to the light/dark cycles on which animals are maintained. The vast majority of studies summarized in this review housed animals on 12 h light/12 h dark cycles. One study kept animals on a 14 h light/10 h dark cycle [44], and two studies did not specify conditions [33,35]. Several studies provided additional details about the timing of experimental procedures [20,24,43]; this information may be relevant to replication efforts. One study empirically compared the effects of normal 12 h light/12 h dark cycles to constant darkness on TBI outcomes [30]. Notably, this study found that results differed depending on whether the animal was kept in constant darkness or not, which suggests that this information should be reported in publications and considered when planning experiments. 

### 3.4. Directions for Future Research

Future research efforts are needed to replicate the effects in published studies. Efforts should focus on dosage optimization and exploration of why the same dose had differing effects across published studies. Efforts to increase generalizability of studies are also needed, such as inclusion of more female test animals as well as more diverse ages. Longer-term histological and behavioral data should be collected. Moreover, better characterization of endogenous changes in the melatonergic system including MEL-specific receptors is needed, both in pre-clinical and clinical studies. Additionally, retrospective comparisons of individuals who were taken melatonin supplements at the time of their injury to those who did not supplement are worth pursuing. Finally, completion of ongoing clinical trials is needed so that more avenues for refinement of the knowledge base can be identified and pursued. 

### 3.5. Limitations in the Methodology Used in This Review

This review, while the most comprehensive of its kind, does have limitations worth noting. Most importantly, only articles originally published in English or with a published English translation were used, which means that important literature from international research teams may not have been included. Likewise, the gray literature (e.g., dissertations) was not searched. A further limitation is the fact that our methods, while rigorous, did not follow any formal guidelines for a systematic review (e.g., the Preferred Reporting Items for Systematic Reviews and Meta-Analyses (PRISMA) guidelines). 

## 4. Materials and Methods

Initial literature searches were conducted in October 2014 and January 2015; at that time, alerts were set to notify the authors of additional publications on the topic of interest. To ensure no new articles were missed, a second search of the literature was conducted in May 2016. During the primary search of the literature, the following web resources were searched: PubMed, PubMed Central, Google Scholar, and the Cochrane Database. The following search terms were used, alone or in combination: traumatic brain injury, TBI, brain injury, head injury, experimental brain injury, Melatonin, MEL, and pineal. Secondary searches were executed using bibliographies from articles identified in the primary search. 

## 5. Conclusions

There are few studies testing MEL therapy after TBI and, with the exception of two clinical trials [52,56], are limited to preclinical models. The results of the 22 pre-clinical studies identified varied, though most found a beneficial effect of MEL at one or more of the dosing regimens trialed. The studies summarized in this review were characterized by variations in the therapeutic regimen (e.g., dose, timing), and outcome variables chosen were largely limited to acute evaluation of cellular and histopathological outcomes. Taken together, the evidence suggests that MEL is a safe and low-toxic drug with neuroprotective properties after TBI. Future studies need to expand the pre-clinical samples to enhance generalizability, as well as examine longer-term histopathological and behavioral outcomes. 

## Appendix A

**Table A1:** Mésenge et al. (1998) Study Details.

**Reference**	Mésenge, C.; Margaill, I.; Verrecchia, C.; Allix, M.; Boulu, R.G.; Plotkine, M. Protective effect of melatonin in a model of traumatic brain injury in mice. *J. Pineal Res.* 1998, *25*, 41–46. PMID: 9694403
**Sample; Groups**	Swiss mice (adult males, weighing 25–30 g). Sample size/groups differed by phase. Groups included: (1) TBI + 1.25 mg/kg MEL given at 20 min pre-TBI, 5 min, 1 h, and 2 h post-TBI; (2) TBI + 1.25 mg/kg MEL at 5 min, 1 h, 2 h, and 3 h post-TBI; (3) TBI + vehicle; (4) TBI + 1.25 mg/kg MEL at 30 min, 1.5 h, 2.5 h, and 3.5 h post-TBI; (5) TBI + 1.25 mg/kg MEL at 1 h, 2 h, 3 h, and 4 h post-TBI; (6) TBI + 0.625 mg/kg MEL at times specified in group 5; (7) TBI + 2.5 mg/kg at times specified in group 5; (8) Uninjured controls (*n* = 15); (9–16) PBN groups with different doses/times (not summarized in table).
**Aim/Hypothesis**	Aim: To compare the effects of melatonin and another drug (α-phenyl-tert-butyl-nitrone [PBN]), a known free radical scavenger, on outcomes of TBI (wire grip & colonic temperature). Hypothesis: Not stated.
**Condition Modeled**	TBI (severity not specified) modeled using weight drop. Non-anesthetized mice were assigned to either the un-injured or injured group. Injured animals were subjected to weight drop using a 50 g weight dropped from a height of 22 cm along a string and onto a metal impounder on the skull. Test animals were sacrificed 24 h after sham or TBI. Some of the injured mice were given vehicle control solution.
**Treatment**	Melatonin was dissolved in HCl and diluted in phosphate buffer to achieve a pH of 7.34. The preparation of PBN is not discussed as MEL is the focus of this review. Both MEL and PBN were administered via 4 i.p. injections. Timing of administration varied and included: (1) 20 min pre-injury, 5 min, 1 h, and 2 h post-TBI (2) 5 min, 1 h, 2 h, and 3 h after TBI (3) 30 min, 1.5 h, 2.5 h, and 3.5 h post-injury (4) 1 h, 2 h, 3 h, and 4 h post-TBI. Dosed by weight: 0.625 mg/kg, 1.25 mg/kg, 2.5 mg/kg, or vehicle.
**Results**	Colonic temperature was not affected by MEL therapy. Wire grip test scores were improved with MEL (1.25 mg/kg given starting 5 min or 20 min pre-injury) vs. TBI + vehicle. The therapeutic window of MEL was limited to an early post-TBI period. Starting MEL at 30 min or later post-TBI was not effective; starting MEL 20 min pre-TBI or 5 min post-TBI was helpful. Dose response of MEL showed a bell-shaped curve with 0.626 and 2.5 mg/kg being ineffective at improving wire grip performance but 1.25 mg/kg effective. The effects of PBN are not summarized due to the scope of this review.
**Conclusion**	To the best of Mésenge et al.’s knowledge, this is the first report to show neuro-protective effects of MEL similar to another free-radical scavenger (PBN). Melatonin reduced neurological deficits after TBI as evidenced by improved wire grip. The therapeutic window of MEL is short and the dose is also an important consideration for future studies.

**Table A2:** Cirak et al. (1999) Study Details.

**Reference**	Cirak, B.; Rousan, N.; Kocak, A.; Palaoglu, O.; Palaoglu, S.; Kilic, K. Melatonin as a free radical scavenger in experimental head trauma. *Pediatr. Neurosurg.* 1999, *31*, 298–301. PMID: 10702729
**Sample; Groups**	Wistar rats (age sex, weight not specified). *n* = 90 rats across 2 experiments. *Stage 1:* 5 groups (*n* = 50): (1) T0: TBI group sacrificed (sac’d) 15 min post-TBI (*n* = 10); (2) T2: TBI & sac’d 2.25 h (*n* = 10); (3) T8: TBI & sac’d 8.25 h (*n* = 10); (4) T 48: TBI & sac’d 48 h (*n* = 10); (5) K: controls (*n* = 10). *Stage 2:* 4 groups: (1) TM0: TBI + 200 mg/kg MEL at time of injury & sac’d 15 min post-TBI (*n* = 10); (2) TM2: TBI + 200 mg/kg MEL at 2 h & sac’d 2.25 h post-TBI (*n* = 10); (3) TM8: TBI + 200 mg/kg MEL at 8 h & sac’d 8.25 h post-TBI (*n* = 10); (4) TM48: TBI + 200 mg/kg MEL at 8 h & sac’d 8.25 h post-TBI (*n* = 10).
**Aim/Hypothesis**	Aim: To test the effects of MEL on outcomes of TBI. Hypothesis: Not specified.
**Condition Modeled**	TBI (severity not specified) modeled using weight drop. Non-anesthetized mice were assigned to either the un-injured or injured group. Injured animals were subjected to weight drop using a 50 g weight dropped from a height of 22 cm along a string and onto a metal impounder on the skull. Test animals were sacrificed 24 h after sham or TBI. Some of the injured mice were given vehicle control solution.
**Treatment**	Melatonin was dissolved in HCl and diluted in phosphate buffer to achieve a pH of 7.34. The preparation of PBN is not discussed as MEL is the focus of this review. Both MEL and PBN were administered via 4 i.p. injections. Timing of administration varied and included: (1) 20 min pre-injury, 5 min, 1 h, and 2 h post-TBI (2) 5 min, 1 h, 2 h, and 3 h after TBI (3) 30 min, 1.5 h, 2.5 h, and 3.5 h post-injury (4) 1 h, 2 h, 3 h, and 4 h post-TBI. Dosed by weight: 0.625 mg/kg, 1.25 mg/kg, 2.5 mg/kg, or vehicle.
**Results**	Colonic temperature was not affected by MEL therapy. Wire grip test scores were improved with MEL (1.25 mg/kg given starting 5 min or 20 min pre-injury) vs. TBI + vehicle. The therapeutic window of MEL was limited to an early post-TBI period. Starting MEL at 30 min or later post-TBI was not effective; starting MEL 20 min pre-TBI or 5 min post-TBI was helpful. Dose response of MEL showed a bell-shaped curve with 0.626 and 2.5 mg/kg being ineffective at improving wire grip performance but 1.25 mg/kg effective. The effects of PBN are not summarized due to the scope of this review.
**Conclusion**	To the best of Mésenge et al.’s knowledge, this is the first report to show neuro-protective effects of MEL similar to another free-radical scavenger (PBN). Melatonin reduced neurological deficits after TBI as evidenced by improved wire grip. The therapeutic window of MEL is short and the dose is also an important consideration for future studies.

**Table A3:** Sarrafzadeh et al. (2000) Study Details.

**Reference**	Sarrafzadeh, A.S.; Thomale, U.W.; Kroppenstedt, S.N.; Unterberg, A.W. Neuroprotective effect of melatonin on cortical impact injury in the rat. *Acta Neurochir.* 2000, *142*, 1293–1299. PMID: 11201646
**Sample; Groups**	Sprague-Dawley rats (adults males with an average weight of 300 g). 4 groups (*n* = 45): (1) TBI + 100 mg/kg MEL for investigation of contusion volume (*n* = 16) with half the rats dosed during the day and half at night); (2) TBI + vehicle (*n* = 15) for investigation of contusion volume; (3) TBI + 100 mg/kg MEL for edema analysis (*n* = 7); (4) TBI + vehicle for edema analysis (*n* = 7). Note: sham controls mentioned but sample size nor details of exposure was not provided.
**Aim/Hypothesis**	Aim: To test the effects of MEL on TBI outcomes (blood gases, contusion volume, blood pressure, edema). Hypothesis was not stated.
**Condition Modeled**	TBI (moderate severity) was induced using the controlled cortical impact model. Anesthetized animals were subjected to sham surgery or controlled cortical impact induced using a pneumatic impactor over the poro-parietal cortex. Injury parameters were as follows: 2 mm depth and 7 m/s velocity. Test animals were humanely euthanized at 24 h post injury.
**Treatment**	Melatonin was dissolved in a 1:10 solution of ethanol in normal saline. Administered via i.p. injection at 4 timepionts: 20 min prior to trauma, immediately after TBI, 1 h after TBI and 2 h after TBI. Dosed by weight 100 mg/kg. Control animals were given ethanoic saline vehicle.
**Results**	Blood gases and pressures were not different across the groups. Contusion volume was reduced when MEL was given at nighttime but not daytime. Hemispheric swelling and edema/water content was not significantly reduced with MEL. Intracranial pressure was not different between the groups.
**Conclusion**	This study shows that MEL significantly reduces contusion volume with major effects during the night, which may be due to reduction of early free radical formation. No other variables were affected by treatment at the dose given. Other doses during daytime and nighttime should be tested

**Table A4:** Beni et al. (2004) Study Details.

**Reference**	Beni, S.M.; Kohen, R.; Reiter, R.J.; Tan, D.-X.; Shohami, E. Melatonin-induced neuroprotection after closed head injury is associated with increased brain antioxidants and attenuated late-phase activation of NF-κB and AP-1. *FASEB J.* 2004, *18*, 149–151. PMID: 14597558
**Sample; Groups**	Sabra mice of the Hebrew University Strain (adult males, weighing 30–42 g). 9 groups (total sample size unclear; groups and respective sample size varied with phase and often presented as a range) *Phase 1: 24 h* (1) Sham (*n* = 5–11 depending on treatment: vehicle, 1 mg/kg, 5 mg/kg, or 10 mg/kg); (2) TBI + vehicle used with MEL (*n* = 10); (3) TBI + 1 mg/kg MEL (*n* = 10); (4) TBI + 5 mg/kg MEL (*n* = 10); (5) TBI + 10 mg/kg MEL (*n* = 10). *Phase 2: 4 or 8 days* (6) TBI + vehicle used with 2-AG (*n* = 5–8); (7) TBI + 5 mg/kg 2-AG (*n* = 5-8); (8) TBI + vehicle used with MEL (*n* = 5–8); (9) TBI + 5 mg/kg MEL (*n* = 5–8).
**Aim/Hypothesis**	Aim: To evaluate the effects of MEL on TBI outcomes (lesion volume, antioxidant profiles and redox-dependent signaling). *Note: 2-arachidonoyl glycerol (2-AG) was also tested. Hypothesis: Not stated.
**Condition Modeled**	TBI (severity not specified) modeled using closed head injury (CHI). Anesthetized mice were immobilized beneath a cylindrical weight drop device and exposed to sham or closed head injury in which a 94g metal rod was dropped from a height of 11–14 cm (depending on the test animal’s weight). Test animals were sacrificed in the acute period (24 h post-injury) or chronic period (4 or 8 days post-injury) depending on the phase of the study.
**Treatment**	MEL was dissolved in a 5% ethanoic saline solution and protected from light until same day use. Administered via a single i.p. injection 1 h after trauma or sham (following 1 h neurological score testing). Dosed by weight: 1 mg/kg; 5 mg/kg; or 10 mg/kg in phase 1 or 5 mg/kg in phase 2 Vehicle groups received MEL vehicle (5% ethanoic saline) or 2-AG vehicle (a 1:1:18 mix of ethanol: chromophore: saline). Note: details of 2-AG preparation, administration & dosing omitted from this table due to scope of review.
**Results**	Neurological severity score (NSS) pre-treatment groups were similar; 5 mg/kg MEL improved NSS at 24 h vs. other groups (*p* = 0.028). MEL mice did better on 7 of 10 tasks and had higher ∆NSS. Lesion size increased after TBI; 2 fold smaller (*p* < 0.01) with MEL vs. vehicle. Brain antioxidants at 1 day after CHI, MEL (5 mg/kg) led to 44% increased antioxidants; no sham effect of MEL. Ascorbic acid- MEL increased ascorbic acid 4 days after CHI; no sham effect. DNA binding activity of NF-κB and AP-1- CHI led to increase in nuclear NF-κB. 5 mg/kg MEL blocked the increase on day 8 but not at other time points examined.
**Conclusion**	This study showed a dose-response effect with 5 mg/kg MEL being effective but not 1 mg/kg or 10 mg/kg. MEL. MEL potentiates post-CHI brain antioxidants, and blocks the late-phase robust activation of NF-κB and decreases AP-1 to half the basal level. Findings suggest MEL exerts effects via ROS scavenging and other nonreceptor-activities. 2-AG treatment was ineffective.

**Table A5:** Ozdemir et al. (2005) Study Details.

**Reference**	Ozdemir, D.; Uysal, N.; Gonenc, S.; Acikgoz, O.; Sonmez, A.; Topcu, A.; Ozdemir, N.; Duman, M.; Semin, I.; Ozkan, H. Effect of melatonin on brain oxidative damage induced by traumatic brain injury in immature rats. *Physiol. Res.* 2005, *54*, 631–637. PMID: 15720160
**Sample; Groups**	Wistar rats (pups aged 7 days post-natal, of unspecified sex). 3 groups (*n* = 21): (1) TBI + vehicle (*n* = 7); (2) TBI + MEL (*n* = 7); (3) Sham control (*n* = 7). Note: it was unclear if the sham controls received vehicle solution.
**Aim/Hypothesis**	Aim: To evaluate the effect of therapeutic MEL on brain antioxidant enzyme activities (e.g, superoxide dismutase [SOD]; glutathione peroxidase [GPx]) and indicators of lipid peroxidation (thiobarbituric acid reactive substances [TBARS]). Hypothesis: Not stated.
**Condition Modeled**	TBI (unspecified severity) modeled using weight drop. Anesthetized test animals were subjected to sham surgery or weight-drop TBI over the parietal bone surface with a force of 160 g·cm produced by 10-g weight which was guided down a 40 cm long tube onto a footplate affixed to the skull. Test animals were sacrificed 24 h after TBI or sham.
**Treatment**	Melatonin dissolved in absolute ethanol and diluted with physiologic saline to a concentration of 5% ethanoic saline. Administered as a single intraperitoneal (i.p) injection immediately after TBI or sham. Dosed by weight (5 mg/kg MEL). Controls given equal volume of 5% ethanoic saline vehicle.
**Results**	TBARS levels expressed as nmol/mg protein were significantly increased (*p* < 0.001) after TBI within both the contralateral and ipsilateral hemisphere compared to controls. MEL treatment after TBI significantly (*p* < 0.001) prevented increase of TBARS in contra- and ipsi-lateral brain, restoring TBARS levels to those seen in sham animals (*p* > 0.05). SOD and GPx levels showed no significant differences across the study groups.
**Conclusion**	To the best of Ozedemir et al.’s knowledge, this is the first publication suggesting MEL reduces lipid per-oxidation after TBI in immature rats. A single dose of 5 mg/kg MEL prevented the increase in TBARS after TBI, suggesting MEL has anti-oxidant properties after head trauma

**Table A6:** Ozdemir et al. (2005) Study Details.

**Reference**	Ozdemir, D.; Tugyan, K.; Uysal, N.; Sonmez, U.; Sonmez, A.; Acikgoz, O.; Ozdemir, N.; Duman, M.; Ozkan, H. Protective effect of melatonin against head trauma-induced hippocampal damage and spatial memory deficits in immature rats. *Neurosci. Lett.* 2005, *385*, 234–239. PMID: 15970378
**Sample; Groups**	Wistar rats (pups of unspecified sex, aged 7 days post-natal) 5 groups (*n* = 63): (1) Control (*n* = 7); (2) Sham untreated (*n* = 14); (3) TBI + vehicle (*n* = 14); (4) TBI + 5 mg/kg MEL (*n* = 14); (5) TBI + 20 mg/kg MEL (*n* = 14).
**Aim/Hypothesis**	Aim: To evaluate if therapeutic MEL after TBI would reduce hippocampal damage and attenuate deficits in spatial memory. Hypothesis: Not stated.
**Condition Modeled**	TBI (unspecified severity) modeled using weight drop. Anesthetized test animals were subjected to sham surgery or weight-drop TBI over the parietal bone surface with a force of 160 g·cm produced by 10-g weight which was guided down a 40 cm long tube onto a footplate affixed to the skull. Test animals were sacrificed at either 24 h or 3 weeks post-TBI or sham (half of each group at each time point).
**Treatment**	Melatonin was dissolved in absolute ethanol and diluted with physiologic saline to a concentration of 5% ethanoic saline. Administered as a single i.p. injection immediately after TBI or sham. Dosed by weight (5 mg/kg MEL or 20 mg/kg MEL). Controls given equal volume of 5% ethanoic saline vehicle.
**Results**	Hippocampal neuron density was reduced in ipsilateral CA1, CA3, & dentate gyrus in TBI + vehicle group vs. sham/control (*p* < 0.01); MEL preserved neurons vs. TBI + vehicle (*p* < 0.01), but not to sham/control levels (*p* > 0.05). Contralateral neuron loss in CA1 and dentate gyrus were attenuated by MEL. TUNEL- positive neurons were detected after injury but less with MEL treatment (*p* < 0.01). Spatial memory: TBI + vehicle rats had longer latencies than sham/control animals (*p* < 0.001). MEL shortened mean latency on 3rd and 4th days of training vs. TBI + vehicle (*p* < 0.01). Probe trial improved with MEL vs. TBI + vehicle (*p* < 0.01).
**Conclusion**	MEL reduced apoptosis and attenuated functional deficits but there was no difference between 5 and 20 mg/kg. Improvement in functional outcome paralleled reduction of cell death. To the best of Ozdemir et al.’s knowledge, this is the first report that MEL improves cognitive outcomes of juvenile TBI. MEL may be a good pediatric TBI therapy.

**Table A7:** Ucar et al. (2005) Study Details.

**Reference**	Ucar, T.; Ozkaya, G.; Demir, N.; Gurer, I.; Akyuz, M.; Onal, M.Z. The effects of environmental light--dark changes on experimental mild traumatic brain injury. *Acta Neurol. Scand.* 2005, *112*, 163–172. PMID: 16097958
**Sample; Groups**	Sprague Dawley rats (adult males weiging 250–360 g). 5 groups (*n* = 56): (1) TBI with normal light/dark cycle (*n* = 14); (2) TBI + 50 mg/kg MEL on a normal light/dark cycle (*n* = 14); (3) TBI + 48 h in constant dark (*n* = 14); (4) TBI + 50 mg/kg MEL + 48 h in constant dark (*n* = 14).
**Aim/Hypothesis**	Aim: To test the effects of normal day/night cycle vs. constant-darkness with or without melatonin therapy on outcomes of TBI. Hypothesis: melatonin secretion after traumatic brain injury would be enhanced by darkness and contribute to neuroprotective effects.
**Condition Modeled**	TBI (mild severity) induced using a modified Marmarou weight drop model. Anesthetized animals were subjected to weight drop injury where a 300 g weight was dropped from a height of 1 m. Test animals were sacrificed 48 h after TBI. Note: There were no sham animals; rather, pre-injury baseline was used.
**Treatment**	Melatonin was dissolved in absolute ethanol and diluted with normal saline to a concentration of 1% ethanoic saline. Administered via a single i.p. injection immediately after TBI. Dosed by weight 50 mg/kg MEL.
**Results**	Motor function was improved in both melatonin groups (regardless of darkness). EEG recordings were not significantly different with MEL therapy (regardless of darkness). Microscopic examination found MEL led to beneficial effects vs. TBI alone. MEL reduced edema in the perivascular and perineuronal regions. MEL preserved neuronal nuclear membranes and euchromatic DNA. MEL also led to healthier neutrophils. Similar effects were seen in the MEL + darkness group though perivascular edema remained. Serum MEL levels only with darkness did MEL therapy raise serum MEL levels.
**Conclusion**	Following mild TBI, darkness with or without MEL lead to neuro-protection. Darkness-induced elevation in endogenous MEL secretion contributes to neuro-protection, though other mechanisms may contribute.

**Table A8:** Ates et al. (2006) Study Details.

**Reference**	Ates, O.; Cayli, S.; Gurses, I.; Yucel, N.; Iraz, M.; Altinoz, E.; Kocak, A.; Yologlu, S. Effect of pinealectomy and melatonin replacement on morphological and biochemical recovery after traumatic brain injury. *Int. J. Dev. Neurosci.* 2006, *24*, 357–363. PMID: 16959465
**Sample; Groups**	Wistar rats (adult males 200–250 g). 6 groups (*n* = 72): (1) craniectomy (crani) for sham group (*n* = 12); (2) crani + TBI (*n* = 12); (3) crani + TBI + 100 mg/kg MEL (*n* = 12); (4) pinealectomy (PX) + crani (*n* = 12); (5) PX + crani + TBI (*n* = 12); (6) PX + TBI + 100 mg/kg MEL (*n* = 12).
**Aim/Hypothesis**	Aim: To examine the effects of TBI with and without pinealectomy (60 days piror to injury) and test the effects of therapeutic melatonin administration. Hypothesis: Not stated.
**Condition Modeled**	TBI (severity not specified) using Marklund’s modified weight drop model. Anesthetized rats were subjected to sham or weight drop injury using a 21 g weight dropped from a height of 35 cm onto a plate resting on the exposed dura. Rats subjected to pinealectomy were allowed to recover for 60 days prior to other study procedures. Test animals were sacrificed at either 24 h or 2 weeks post TBI or sham.
**Treatment**	Melatonin preparation was not specified. Administered via a single i.p. injection immediately after injury. Dosed by weight 100 mg/kg MEL. No vehicle control solution was given.
**Results**	Malondialdehyde (MDA) levels were elevated with PX vs. control or TBI in non-PX rats. MEL decreased MDA more in TBI than PX + TBI. Glutathione (GSH) levels were lower in PX rats than control, TBI reduced GSH; MEL increased GSH. Nitric Oxide (NO) levels were elevated after PX vs. control rats. MEL after TBI in both PX and non-PX rats reduced NO levels. Xanthine oxidase (XO) levels increased in PX rats vs. control rats. XO levels were reduced in both MEL groups. Histopathological lesion was decreased by MEL, larger decrease in trauma alone group than in group with PX + TBI.
**Conclusion**	This study was the first to explore the effects of PX and MEL therapy in the context of TBI. PX and TBI alone and in combination caused oxidative stress that was prevented with MEL therapy. High-dose MEL immediately post-TBI is neuro-protective in this TBI model and warrants further study.

**Table A9:** Jadha et al. (2009) Study Details.

**Reference**	Jadhav, V.; Lee, S.; Ayer, R.E.; Rojas, H.; Hyong, A.; Lekic, T.; Tang, J.; Zhang, J.H. Dual effects of melatonin on oxidative stress after surgical brain injury in rats. *J. Pineal Res.* 2009, *46*, 43–48. PMID: 18573160
**Sample; Groups**	Sprague-Dawley rats (adult males, weighing 200–350 g). 5 groups (total sample size unclear and group sizes depend on outcome): (1) Sham; (2) TBI + vehicle; (3) TBI + 5 mg/kg MEL; (4) TBI + 15 mg/kg MEL; (5) TBI + 150 mg/kg MEL.
**Aim/Hypothesis**	Aim: To test the effects of surgical brain injury on oxidative stress, edema, and neurological outcomes. Hypothesis: MEL will decrease oxidative stress and attenuate postoperative complications (e.g., edema; neurological deficits).
**Condition Modeled**	TBI (severity not specified) modeled using surgical brain injury (SBI). Anesthetized test animals were subjected to sham (craniotomy + bone flap replacement) or SBI (right dorsum incision, blunt dissection of skin/ connective tissue, a 5 mm square craniectomy made using a drill such that the lower left edge was at bregma, incision into the underlying dura exposed the right frontal lobe, incisions made, a piece of brain tissue resected; hemostasis was achieved using intraoperative packing and saline irrigation and the bone flap replaced and skin sutured). Test animals were sacrificed 24 h post-TBI or sham.
**Treatment**	Melatonin was dissolved in a mixture of 10% ethanoic saline. Administered as a single i.p. injection 1 h before surgery. Dosed by weight (5 mg/kg MEL, 15 mg/kg MEL, and 150 mg/kg MEL). Control-treated mice received an equal volume of 10% ethanoic saline vehicle.
**Results**	Edema (brain water content) was increased after SBI (vs. sham) and not attenuated by MEL 150 mg/kg dose increased edema. Lipid peroxidation (malondialdehyde [MDA] levels); TBI + vehicle had 6× peroxidation vs. sham; 15 mg/kg MEL reduced oxidative stress, but 150 mg/kg increased it. Composite neuro-score worsened after SBI with or without MEL (150 mg/kg worsened). Vibrissae stimulation study (VSS):15mg/kg dose improves VSS but 150 mg/kg dose made worse. Beam balance test: showed mixed deficits of SBI and no treatment effect of MEL with some detrimental effects.
**Conclusion**	Overall, the study suggested duel effects of MEL: low doses reduced oxidative stress and protect the brain; high doses, however, have deleterious effects including increased edema, peroxidation and worsened neurological outcomes. The safety of high-dose MEL must be further tested.

**Table A10:** Kabadi et al. (2010) Study Details.

**Reference**	Kabadi, S.V.; Maher, T.J. Posttreatment with uridine and melatonin following traumatic brain injury reduces edema in various brain regions in rats. *Ann. N. Y. Acad. Sci.* 2010, *1199*, 105–113. PMID: 20633115
**Sample; Groups**	Sprague-Dawley rats (adult males, weighing 300–350 g). 4 groups (sample size unclear; groups depend study phase): *Phase 1 = 1 drug only* (1) Sham + vehicle (V) (*n* = 12); (2) Sham + drug: given either 23 mg/kg uridine [UR] or 200 mg/kg MEL (*n* = 6); (3) TBI + V (*n* = 12); (4) TBI + drug: either 16 or 32 mg/kg UR or 100 or 200 mg/kg MEL (*n* = 8–10). *Phase 2 = 2 drugs* (1) Sham + V (*n* = 9); (2) Sham + drug: given 32 mg/kg UR + 200 mg/kg MEL (*n* = 6); (3) TBI + V (*n* = 9); (4) TBI + drug: given combination of 16 mg/kg UR + 200 mg/kg MEL or 32 mg/kg UR + 200 mg/kg MEL (*n* = 7–8).
**Aim/Hypothesis**	Aim: To test the effects of treatment with melatonin and/or uridine on edema after TBI. Hypothesis: MEL and uridine therapy, alone and in combination, will attenuate edema after TBI induced using the fluid percussion injury (FPI) model.
**Condition Modeled**	TBI (severity not specified) modeled using lateral FPI. Anesthetized test animals were subjected to right side craniectomy and FPI (pressure of 2.5–2.8 atms). Test animals were sacrificed 48 h post-TBI or sham.
**Treatment**	Melatonin was dissolved in polyethylene glycol (PEG) 400 in a 1:1 (*v*/*v*) solution with sterile water. Uridine was dissolved in saline. Melatonin and/or uridine was administered as a single i.p. injection 15 min post-injury. Dosed by weight (1 mL/kg of uridine for a total dose of 16 mg/kg or 32 mg/kg and/or 2 mL/kg of MEL for a total dose of 100 mg/kg or 200 mg/kg). Control-treated rats received an equal volume of vehicle.
**Results**	Edema (wet/dry weight) was observed in the ipsilateral cortex, hippocampus, and striatum after FPI vs. sham. Both doses of uridine reduced edema (*p* < 0.05) in the striatum (vs. TBI + vehicle). High dose MEL (200 mg/kg) reduced edema in the striatum (*p* < 0.05) vs. TBI + vehicle. No significant edema on the contralateral side, nor was there any treatment effect. Both combination treatments failed to reduce ipsilateral cortical edema but reduced (*p* < 0.05) hippocampal and striatal edema. On the contralateral side, both combinations reduced (*p* < 0.05) striatal edema but only the high-dose uridine with high-dose MEL had less hippocampal edema. No treatment effect in sham groups was reported.
**Conclusion**	First study to report treatment with uridine and MEL (alone and in combination) reduce edema after TBI. It is important to note that some of the effects of uridine may be due to its hypothermic effects. Moreover, since MEL and uridine have different actions, there may be more than one mechanism underlying the effects observed.

**Table A11:** Kelso et al. (2011) Study Details.

**Reference**	Kelso, M.L.; Scheff, N.N.; Scheff, S.W.; Pauly, J.R. Melatonin and minocycline for combinatorial therapy to improve functional and histopathological deficits following traumatic brain injury. *Neurosci. Lett.* 2011, *488*, 60–64. PMID: 21056621
**Sample; Groups**	Sprague-Dawley rats (adult males, weighing 225–275 g). 4 groups (*n* = 50) in each of 2 experiments: animals were either sacrificed 12 days post-injury (experiment 1) or 7 days post-injury (experiment 2). *Experiment 1 (n = 31):* (1) TBI + vehicle (*n* = 6–9: exact group sizes not specified); (2) TBI + melatonin (*n* = 6–9); (3) TBI + minocycline (*n* = 6-9); (4) TBI + melatonin + minocycline (*n* = 6–9). *Experiment 2 (n* = *19):* (1) TBI + vehicle (*n* = 6–9); (2) TBI + melatonin (*n* = 6–9); (3) TBI + minocycline (*n* = 6–9).
**Aim/Hypothesis**	Aim: To test the effects of MEL and minocycline therapy on outcomes of TBI (cognitive outcomes assessed using Morris water maze, cortical tissue sparing, and ^3^H-PK11195 autoradiography. Hypothesis: combination therapy with MEL and minocycline will be more effective than MEL or minocycline alone.
**Condition Modeled**	TBI (severity not specified) modeled using the controlled cortical impact (CCI). Anesthetized animals were subjected to sham or TBI using CCI (5 mm impactor diameter, 3.5 m/s velocity, 400 ms dwell time) with a depth of 1.5 mm in experiment 1 or 2.0 mm in experiment 2. Test animals were sacrificed after cognitive testing completion on either day 12 or 7 post-TBI.
**Treatment**	Melatonin and/or minocycline were dissolved in a solution of 2% ethanol in phosphate buffered saline. Administered via 2 i.p. injections. Dosing schedule depended on drug and experiment. *Experiment 1:* MEL administered at 5 and 60 min post injury. Minocycline (MIN) was administered on days 1–4 post-injury. *Experiment 2* MEL and MIN were administered at 5 min and 90 min post-injury. Dosed by weight depending on experiment. *Experiment 1:* MEL = 5 mg/kg. MIN = 40 mg/kg. *Experiment 2:* MEL = 5 mg/kg. MIN = 45 mg/kg.
**Results**	Cognitive assessment was not significantly different across the groups. Cortical tissue sparing was not significantly different across any of the groups. ^3^H-PK11195 binding was not significantly different across the groups.
**Conclusion**	There was no neuro-protective effect of MEL and/or minocycline after TBI in this study. More focal nature of CCI vs. weight drop may have led to less pineal damage and thus less effect of exogenous MEL due to endogenous release. Other studies have showed beneficial effects, suggesting additional research is needed.

**Table A12:** Dehghan et al. (2013) Study Details.

**Reference**	Dehghan, F.; Khaksari Hadad, M.; Asadikram, G.; Najafipour, H.; Shahrokhi, N. Effect of melatonin on intracranial pressure and brain edema following traumatic brain injury: Role of oxidative stresses. *Arch. Med. Res.* 2013, *44*, 251–258. PMID: 23608674
**Sample; Groups**	N-Mary rats (adult males, weighing 250–300 g). 5 groups (*n* = 140): (1) Sham (*n* = 28); (2) TBI (*n* = 28); (3) TBI + vehicle (*n* = 28); (4) TBI + 5 mg/kg MEL (*n* = 28); (5) TBI + 20 mg/kg MEL (*n* = 28). Note: The 5 groups of rats divided into 4 subgroups (*n* = 7) to examine different endpoints: Subgroup 1: brain water content + neuro-score; Subgroup 2: Blood-brain-barrier (BBB) permeability via Evans blue dye content; Subgroup 3: intra-cranial pressure (ICP); Subgroup 4: oxidant parameters, including malondialdehyde (MDA), glutathione peroxidase (GPx), Superoxide Dismutase (SOD).
**Aim/Hypothesis**	Aim: To study the effect of MEL on TBI outcomes (edema; ICP; neurological outcome) at different post-injury time points. Hypothesis: Not stated.
**Condition Modeled**	TBI (severity not specified) modeled using Marmaou’s weight drop model. Anesthetized test animals were subjected to sham surgery or weight drop injury where TBI was produced by dropping a 250 g weight onto a steel disk affixed to the skull from a height of 2 m. Test animals were sacrificed 72 h post-TBI or sham. Approximately 25% mortality rate occurred in this study.
**Treatment**	Melatonin was prepared in an ethanoic saline solution (concentration of ethanol was not specified). Administered via 4 i.p. injections given at 1 h, 24 h, 48 h, and 72 h post-injury. Dosed by weight: 5 mg/kg or 20 mg/kg. Control-treated rats received a constant volume of ethanoic saline vehicle (0.33 mL/rat).
**Results**	Edema higher after TBI; both MEL doses reduced edema vs. TBI/TBI + vehicle. BBB Permeability- Evans blue content higher after TBI, MEL groups had lower content than TBI/TBI + vehicle. ICP increased 1 h post-TBI and was reduced with both MEL doses at 24, 48, and 72 h post-injury. GPx and SOD levels increased in MEL groups vs. TBI/TBI + vehicle. (MDA) Level- at 72 h TBI increased MDA levels vs. sham which was lowered by both MEL doses. Veterinary Coma Scale scores at 1 h all TBI groups did worse than sham (*p* < 0.001). At 24, 48, and 72 h, both doses had better scores than TBI/TBI + vehicle.
**Conclusion**	Low- and high- dose MEL decreased brain edema and BBB permeability at 72 h after TBI; MEL also improved neurologic scores and ICP. The neuro-protective effects reported may be due to MEL increasing antioxidant enzymes and decreasing oxidant agents (e.g., free radicals). ICP probe insertion could have contributed to elevated ICP.

**Table A13:** Campolo et al. (2013) Study Details.

**Reference**	Campolo, M.; Ahmad, A.; Crupi, R.; Impellizzeri, D.; Morabito, R.; Esposito, E.; Cuzzocrea, S. Combination therapy with melatonin and dexamethasone in a mouse model of traumatic brain injury. *J. Endocrinol.* 2013, *217*, 291–301. PMID: 23532863
**Sample; Groups**	CD1 mice (adult males, weighing 25–30 g and aged 10–12 weeks). 5 groups (*n* = 50): (1) Sham + vehicle (*n* = 10); (2) TBI + vehicle (*n* = 10); (3) TBI + 0.025 mg/kg dexamethasone (DEX) (*n* = 10); (4) TBI + 10 mg/kg MEL (*n* = 10); (5) TBI + 10 mg/kg MEL + 0.025 mg/kg DEX (*n* = 10)
**Aim/Hypothesis**	Aim: To test the effects of MEL and/or DEX therapy on outcomes of TBI (rotarod test, elevated body swing task, TTC staining, metalloproteinase expression, apoptosis, iNOS expression, and histology). Hypothesis: Not stated.
**Condition Modeled**	TBI (severity not specified) modeled using controlled cortical impact (CCI). Anesthetized mice were subjected to sham or CCI (4 mm tip diameter, 1.5 m/s velocity, unspecified dwell time) with a depth of 3 mm. Test animals were sacrificed 24 h after sham or TBI.
**Treatment**	Melatonin was dissolved in a solution of 1% ethanoic saline. Both MEL and DEX were administered via two i.p. injections at 1 h and 6 h post-TBI. Dosed by weight depending on drug. MEL = 10 mg/kg. DEX = 0.025 mg/kg. Sham animals were given 1% ethanoic saline.
**Results**	Motor function modestly improved with MEL or DEX; MEL + DEX led to greater improvement. Histology revealed damage (gliosis, inflammation, thick vessels); neither MEL nor DEX improved histology; MEL + DEX therapy did. Infarction volume was examined using TTC staining which was attenuated by MEL + DEX not either drug alone. Metalloproteinase expression after TBI was reduced by both therapies alone and even further reduced by combination. iNOS expression was reduced by both MEL or DEX alone; more so with MEL + DEX. Apoptosis was reduced by both MEL or DEX alone; more so with MEL + DEX.
**Conclusion**	MEL + DEX or other multi-drug therapy may be necessary to effectively manage TBI. Combining MEL and DEX therapy has beneficial effects after TBI.

**Table A14:** Senol et al. (2014) Study Details.

**Reference**	Senol, N.; Nazıroğlu, M. Melatonin reduces traumatic brain injury-induced oxidative stress in the cerebral cortex and blood of rats. *Neural Regener. Res.* 2014, *9*, 1112–1116. PMID: 25206769
**Sample; Groups**	Sprague-Dawley rats (6 mo males weighing 300–340 g). 4 groups (*n* = 32): (1) Control + vehicle (*n* = 8); (2) Control + 10 mg/kg MEL (*n* = 8); (3) TBI only (*n* = 8); (4) TBI + 5 mg/kg MEL (*n* = 8).
**Aim/Hypothesis**	Aim: To evaluate the effect of MEL on TBI outcomes (oxidative stress; antioxidant levels). Hypothesis: Melatonin will modulate oxidative stress and may increase antioxidant levels
**Condition Modeled**	TBI (severity not specified) modeled using Marmarou’s weight drop model. Anesthetized animals were subjected to sham surgery or weight drop injury where TBI was produced by dropping a 300 g weight onto a steel disk affixed to the skull from a height of 2 m. Test animals were sacrificed 24 h after MEL administration.
**Treatment**	Melatonin was dissolved in 0.1 mL ethanol and diluted with 0.9 mL isotonic saline for a 0.9% *v*/*w* concentration. Administered via i.p. injection 1 h after trauma or control. Dosed by weight (10 mg/kg in Control + MEL group and 5 mg/kg in TBI + MEL group). Vehicle treated rats were give a constant volume of ethanoic saline vehicle (1 mL/rat).
**Results**	Lipid peroxidation levels in the cerebral cortex, plasma, and erythrocytes were elevated in the TBI group vs. controls. TBI + MEL therapy lowered peroxidation levels (vs. TBI). Glutathione peroxidase (GPx) activity & glutathione level; GPx activity was lower after TBI vs. control; activity did not differ between TBI and TBI + MEL groups. Antioxidant vitamin levels- vitamin A and E levels in the cortex and plasma were not affected by TBI or MEL therapy. β-carotene, vitamin C and vitamin E levels in the cortex were lower after TBI vs. control. Cerebral cortex β-carotene and vitamin C levels and plasma vitamin C level were increased in the TBI + MEL group vs. TBI alone.
**Conclusion**	In this study, MEL protected against peroxidation caused by TBI and promoted antioxidant activity. MEL has protective effects and may be useful as a TBI therapeutic once better studied. To the best of the Senol et al.’s knowledge, this is the first publication to test MEL’s effect on oxidative stress/ antioxidants after TBI in rats.

**Table A15:** Yürüker et al. (2015) Study Details.

**Reference**	Yürüker, V.; Naz, M.; Nilgün, Ş. Reduction in traumatic brain injury-induced oxidative stress, apoptosis, and calcium entry in rat hippocampus by melatonin: Possible involvement of TRPM2 channels. *Metab. Brain Dis.* 2015, *30*, 223–231. PMID: 25339252
**Sample; Groups**	Sprague-Dawley rats (adult males, weighing 340–360 g) 4 groups (*n* = 32): (1) control + vehicle control (*n* = 8); (2) control + 5 mg/kg MEL (*n* = 8); (3) TBI + vehicle (*n* = 10); (4) TBI + 6 mg/kg MEL (*n* = 10).
**Aim/Hypothesis**	Aim: To test the effect of MEL on outcomes of TBI (oxidative stress, apoptosis, and calcium entry through TRPM2 channels). Hypothesis was not stated.
**Condition Modeled**	TBI (unspecified severity) was induced using the Marmarou weight drop method. Anesthetized rats were subjected to sham or weight drop injury where a 300 g weight was dropped onto a steel disk on the head from a height of 2 m. Test animals were sacrificed 24 h after melatonin treatment, which was administered 1 h post injury. Note: there was no sham group.
**Treatment**	Melatonin was dissolved in 0.1 mL of ethanol and diluted with physiologic saline to a volume of 1 mL. Administered via a single i.p. injection 1 h after brain trauma. Dosed by weight 5 mg/MEL. Vehicle control animals were given ethanoic saline vehicle.
**Results**	Calcium (Ca^2+^) concentration was higher (*p* < 0.001) in the TBI group than in the control and MEL groups. MEL reduced Ca^2+^ entry into hippocampal neurons, likely through TRPM2 channels. Apoptosis (caspase-3, caspase-3) was elevated after TBI vs. control and MEL groups, but lower with TBI + MEL than control and MEL groups. Mitochondrial membrane depolarization and intracellular ROS production was higher after TBI when compared to control + MEL groups; TBI + MEL had lower ROS values than TBI + vehicle. TBI + MEL was associated with higher mitochondrial membrane depolarization than in the control group.
**Conclusion**	A significant protective effect of melatonin on Ca^2+^ homeostasis in hippocampal neurons was reported. MEL therapy may prevent activation of TRMP2 channels after TBI and provide benefit for TBI outcomes.

**Table A16:** Ding et al. (2015) Study Details.

**Reference**	Ding, K.; Xu, J.; Wang, H.; Zhang, L.; Wu, Y.; Li, T. Melatonin protects the brain from apoptosis by enhancement of autophagy after traumatic brain injury in mice. *Neurochem. Int.* 2015, *91*, 46–54. PMID: 26527380
**Sample; Groups**	CD1 mice (adult males, weighing 28–32 g). Both Nrf-2 wild-type and knock out mice were used. 6 groups (total sample size not specified): (1) Sham (*n* = 6 per assessment); (2) TBI (*n* = 6 per assessment); (3) TBI + saline (*n* = 6 per assessment); (4) TBI + melatonin (*n* = 6 per assessment); (5) TBI + 3-methyladenine (3-MA) (*n* = 6 per assessment); (6) TBI + melatonin + 3-MA (*n* = 6 per assessment).
**Aim/Hypothesis**	Aim: To evaluate the effects of melatonin on outcomes of TBI (autophagy, apoptosis, and other markers of secondary brain injury). Hypothesis: Not stated.
**Condition Modeled**	TBI (severity not specified) was induced using a Marmarou weight drop model. Anesthetized animals were subjected to sham or weight drop injury where a 200 g weight was dropped onto a disk on the skull; the scalp was sutured closed. Test animals were sacrificed 24 h after TBI for histological analysis or at 7 days for neurological testing.
**Treatment**	Melatonin was dissolved in 5% ethanoic saline and 3-MA was dissolved in saline. Melatonin was administered via 5 i.p. injections at 0 h, 1 h, 2 h, 3 h, and 4 h post-TBI or sham. 3-MA was administered in 1 intra-cerebroventricular injection 15 min prior to TBI. Dosed by weight: 10 mg/kg MEL Control animals received an equal volume saline.
**Results**	Neurological severity score was improved with MEL treatment at 1 and 3 days post-TBI but not 7 days post-TBI. Autophagy appeared to be activated with MEL therapy. Apoptosis was decreased with melatonin treatment, which was reversed with 3-MA treatment.
**Conclusion**	MEL ameliorated secondary brain injury and enhanced autophagy. 3-MA reversed the beneficial effects of MEL in this study.

**Table A17:** Babaee et al. (2015) Study Details.

**Reference**	Babaee, A.; Eftekhar-Vaghefi, S.H.; Asadi-Shekaari, M.; Shahrokhi, N.; Soltani, S.D.; Malekpour-Afshar, R.; Basiri, M. Melatonin treatment reduces astrogliosis and apoptosis in rats with traumatic brain injury. *Iran. J. Basic Med. Sci.* 2015, *18*, 867–872. PMID: 26523219
**Sample; Groups**	NMRI rats (adult males, weighing 250–300 g). 5 groups (*n* = 40): (1) Sham + vehicle (*n* = 8); (2) TBI + vehicle (*n* = 8); (3) TBI + 5 mg/kg MEL (*n* = 8); (4) TBI + 10 mg/kg MEL (*n* = 8); (5) TBI + 20 mg/kg MEL (*n* = 8).
**Aim/Hypothesis**	Aim: To examine the effects of melatonin on outcomes of TBI (apoptosis and astrocyte activation). Hypothesis: Not stated.
**Condition Modeled**	TBI (moderate severity) was induced using a Marmarou weight drop model. Anesthetized animals were subjected to sham or weight drop injury where a 250 g weight was dropped onto a steel disk affixed to the skull from a height of 2 m. Test animals were sacrificed 72 h after TBI or sham.
**Treatment**	Melatonin was dissolved in 1% ethanoic saline. Administered via 4 i.p. injections at 1 h, 24 h, 48 h, and 72 h post-TBI or sham. Dosed by weight: 5 mg/kg, 10 mg/kg or 20 mg/kg. Control animals received ethanoic saline vehicle.
**Results**	Apoptosis was elevated after TBI and reduced with MEL therapy; all 3 doses were effective in preserving neurons compared to vehicle (*p* < 0.05). Activated astrocytes were increased after TBI as evidenced by a higher number of GFAP-positive astrocytes; MEL reduced activated astrocytes but did not show dose-dependent effects.
**Conclusion**	MEL therapy diminished apoptosis and astrocyte reactivity. However, no dose-dependent effects were found. Mechanisms by which melatonin reduces GFAP-positive astrocytes remain to be elucidated as do the chronic effects of therapy.

**Table A18:** Shochat et al. (2015) Study Details.

**Reference**	Shochat, A.; Abookasis, D. Differential effects of early postinjury treatment with neuroprotective drugs in a mouse model using diffuse reflectance spectroscopy. *Neurophotonics* 2015, *2*, 015001. PMID: 26157981
**Sample; Groups**	ICR mice (adult males, weight approximately 40 g and approximately 12 weeks old). 6 groups (*n* = 60): (1) TBI alone (*n* = 10); (2) TBI + hypertonic saline (*n* = 10); (3) TBI + morphine (*n* = 10); (4) TBI + mannitol (*n* = 10); (5) TBI + melatonin (*n* = 10); (6) TBI + minocycline (*n* = 10). Note: The paper also mentions a sham group (*n* = 10) that does not appear to be included in the final sample size of 60 listed in the paper.
**Aim/Hypothesis**	Aim was two-fold: (1) To test the effects of 5 different drugs (hypertonic saline, morphine, mannitol, melatonin, and minocycline) on TBI outcomes; (2) To identify the most promising drug n this injury model. Hypothesis: Not stated.
**Condition Modeled**	TBI (severity not specified) induced using a closed head weight drop model. Anesthetized mice were subjected to sham or weight drop injury where a 50 g cylindrical rod was dropped from a height of 90 cm onto the mouse’s intact skull. Test animals were sacrificed 50 min after TBI or sham (30 min after therapy was administered).
**Treatment**	Melatonin preparation was not described in detail. Administered via a single i.p. injection at 20 min post-TBI. Dosed by weight: 10 mg/kg MEL. Note: Details regarding hypertonic saline, mannitol, morphine, and minocycline are not the subject of this review and are not included in this table.
**Results**	Melatonin had a significant effect on TBI outcomes vs. injury without treatment; specifically MEL decreased HbO_2_ and StO_2_, stabilized Hbr and hemodynamics, and decreased THC. The end result was that MEL reduced the extent of further brain damage. The results of the other drugs are not summarized in this table given the scope of this review focuses on MEL therapy.
**Conclusion**	To the best of Shochat et al.’s knowledge, this study is the first to show macroscopic recovery of hemodynamic and morphologic parameters testing the effects of 5 drugs. Melatonin was not the most effective drug tested.

**Table A19:** Kelestemur et al. (2016) Study Details.

**Reference**	Kelestemur, T.; Yulug, B.; Caglayan, A.B.; Beker, M.C.; Kilic, U.; Caglayan, B.; Yalcin, E.; Gundogdu, R.Z.; Kilic, E. Targeting different pathophysiological events after traumatic brain injury in mice: Role of melatonin and memantine. *Neurosci. Lett.* 2016, *612*, 92–97. PMID: 26639427
**Sample; Groups**	BALB/c mice (adult males, 23–25 g). 4 groups (*n* = 30): (1) TBI + vehicle (*n* = 7); (2) TBI + 4 mg/kg MEL (*n* = 8); (3) TBI + 20 mg/kg memantine (*n* = 8); (4) TBI + 4 mg/kg MEL + 20 mg/kg memantine (*n* = 7).
**Aim/Hypothesis**	Aim: To test the effects of melatonin and memantine alone and in combination on outcomes of TBI (DNA fragmentation; intracellular signaling; infarct volume). Hypothesis: Not stated.
**Condition Modeled**	TBI (severity not specified) was induced using a cold injury model. Anesthetized animals were placed in a stereotaxic device, a craniectomy made, and a liquid nitrogen cooled copper probe (2.5 mm in diameter) paced onto the dura for 60 s before the scalp was sutured closed. Test animals were sacrificed 24 h after TBI
**Treatment**	Melatonin was dissolved in 5% ethanoic saline. Administered via a single i.p. injection immediately after injury induction. Dosed by weight: 4 mg/kg MEL and/or 20 mg/kg memantine. Control animals were given 100 microliters of 5% ethanoic saline.
**Results**	Brain infarct volume was significantly reduced by combination therapy but not either drug alone. DNA fragmentation was decreased with MEL, memantine, and combination. Intracellular signaling after TBI was affected by MEL and memantine alone increasing phosphorylation of JNK1/ERK-1/2 and reducing iNOS activity. Combination therapy reversed the effect of MEL & memantine alone.
**Conclusion**	In this study, a combination of melatonin and memantine was especially effective in improving TBI outcomes. Further pre-clinical and clinical studies are warranted.

**Table A20:** Lin et al. (2016) Study Details.

**Reference**	Lin, C.; Chao, H.; Li, Z.; Xu, X.; Liu, Y.; Hou, L.; Liu, N.; Ji, J. Melatonin Attenuates Traumatic Brain Injury-induced Inflammation: A Possible Role for Mitophagy. *J. Pineal Res*. 2016, *61*, 177–186. PMID: 27117839
**Sample; Groups**	Sprague-Dawley rats (adult males, weighing 220–250 g, aged 8 weeks). The total sample size was unclear, and made up of 5 groups with 5–12 rats per group as follows: (1) Sham; (2) TBI + vehicle; (3) TBI + mitphagy inhibitor; (4) TBI + MEL; (5) TBI + mitophagy inhibitor + MEL.
**Aim/Hypothesis**	Aim: To test the effects of MEL on outcomes of TBI (inflammation, immuno-histochemistry, cell death, edema, lesion volume, motor function, cognitive function). Hypothesis: MEL may ameliorate release of pro-inflammatory cytokines after TBI.
**Condition Modeled**	TBI (severity not specified) modeled using the controlled cortical impact (CCI) model. Anesthetized rats were exposed to sham surgery or CCI using a 6 mm metal impactor tip to induce injury using the following parameters: 2.5 mm depth, 50 ms duration, and 6 m/s velocity. Test animals were sacrificed at 8 h post-TBI/sham for cellular analysis or 16 days post-TBI/sham for behavioral analysis.
**Treatment**	Melatonin was dissolved in 2% ethanoic saline. Administered via i.p. injection at 3 timepionts: 0 h, 2 h, and 4 h post-TBI. Dosed by volume: 5 mL/kg with exact amount of MEL used to prepare the solution not specified. Control animals were given ethanoic saline vehicle. Details of the preparation, administration, and dosing of the mitophagy-inhibitors are not summarized in this table.
**Results**	Post-TBI secretion of inflammatory molecules (IL-1β, IL-6, IL-18, caspase 1, and mature IL-1β) was attenuated by MEL. Apoptotic neuronal death was reduced with MEL therapy. Edema was significantly reduced with MEL therapy. Lesion volume was significantly reduced with MEL therapy. Motor function on the Beam Balance Task was improved with MEL therapy. Note: Some of the effects of MEL were reversed with co-treatment with mitophagy inhibitor. Cognitive outcomes on the Morris water maze were improved with MEL.
**Conclusion**	Melatonin had many protective effects in this study, some of which were reversed with mitophagy inhibitors. Thus, this study suggests melatonin’s beneficial effects are partly related to mitophagy.

**Table A21:** Alluri et al. (2016) Study Details.

**Reference**	Alluri, H.; Wilson, R.L.; Anasooya Shaji, C.; Wiggins-Dohlvik, K.; Patel, S.; Liu, Y.; Peng, X.; Beeram, M.R.; Davis, M.L.; Huang, J.H.; et al. Melatonin Preserves Blood-Brain Barrier Integrity and Permeability via Matrix Metalloproteinase-9 Inhibition. *PLoS ONE* 2016, *11*, e0154427. PMID: 27152411
**Sample; Groups**	C57BL/6 mice (adults of unspecified sex, weighing 25–30 g). 4 groups (*n* = 23): (1) Sham (*n* = 6); (2) Sham + vehicle (*n* = 6); (3) TBI + vehicle (*n* = 5); (4) TBI + 10 mg/kg MEL (*n* = 6). Note: cell culture was also used in this study but is not the focus of this review. (5) TBI + mitophagy inhibitor + MEL.
**Aim/Hypothesis**	Aim: To test the effects of melatonin on blood-brain-barrier breakdown after TBI. Hypothesis: Not stated.
**Condition Modeled**	TBI (mild severity) was induced using the controlled cortical impact (CCI) model. Anesthetized animals were subjected to sham surgery (craniectomy) or TBI induced using a pneumatic CCI device with the 3mm diameter impactor tip contacting the brain between lambda and bregma with the following injury parameters: 2 mm depth, 100 ms duration, and 0.5 m/s velocity. Test animals were humanely euthanized at 24 h post injury.
**Treatment**	Melatonin was dissolved in a solution of alcohol (50 μg/μL) and diluted in Evans blue. Administered via a single intravenous (i.v.) injection (into the tail vein) and allowed to circulate in the animal for 30 min prior to injury (or sham) induction. Dosed by weight: 10 mg/kg. Note: Other drugs were tested in the cell culture portion of this study but are not the focus of this review.
**Results**	Blood-brain-barrier permeability was increased after TBI as evidenced by Evans blue leakage. MEL attenuated blood-brain-barrier permeability. Note: Some of the effects of MEL were reversed with co-treatment with mitophagy inhibitor. Cognitive outcomes on the Morris water maze were improved with MEL.
**Conclusion**	MEL represents a potential therapeutic for reducing blood-brain-barrier permeability after TBI. The beneficial effects in vivo were also paralleled in the in vitro component of the study (not described in detail in this table).

**Table A22:** Wu et al. (2016) Study Details.

**Reference**	Wu, H.; Shao, A.; Zhao, M.; Chen, S.; Yu, J.; Zhou, J.; Liang, F.; Shi, L.; Dixon, B.J.; Wang, Z.; et al.; Melatonin attenuates neuronal apoptosis through up-regulation of K^+^–Cl^−^ Cotransporter KCC2 expression following traumatic brain injury in rats. *J. Pineal Res.* 2016. PMID: 27159133
**Sample; Groups**	Sprague-Dawley rats (adult males, weighing 300–330 g). A total sample of *n* = 156 was divided across two experiments. *Experiment 1:* 7 groups (*n* = 84) divided into 7 post-injury sacrifice groups to examine the time course of K^+^-Cl^−^ cotransporter-2 (KCC2) expression (details omitted per focus of review). *Experiment 2:* 3 groups (*n* = 72): (1) Sham + vehicle (*n* = 24); (2) TBI + vehicle (*n* = 24); (3) TBI + 10 mg/kg MEL (*n* = 24).
**Aim/Hypothesis**	Aim: To test the effects of MEL on neuron-specific KCC2 expression and apoptosis. Hypothesis: Not stated.
**Condition Modeled**	TBI (severity not specified) was induced using the controlled cortical impact (CCI) model. Anesthetized animals were subjected to sham surgery (craniectomy) or TBI induced using an electromagnetic CCI device with a 4 mm diameter tip and the following parameters: 2 mm depth, 120 ms duration, and 3 m/s velocity. Test animals were humanely euthanized at 24 h post-injury (Note: in experiment 1 the time-course of KCC2 was studied out to 168 h post-TBI, but there was no MEL therapy in this portion of the study).
**Treatment**	Melatonin was dissolved in 1 mL of 1% ethanoic saline (vehicle). Administered via 5 i.p. injections at the following time points post-TBI or sham induction: 5 min, 1 h, 2 h, 3 h, and 4 h. Dosed by weight: 10 mg/kg.
**Results**	Cortical neuron degeneration (fluoro-jade B staining) was increased after TBI (vs. sham) and attenuated by MEL therapy (*p* < 0.05). Brain water content was increased by TBI and significantly reduced (*p* < 0.05) by MEL therapy. Neurological deficits using a modified neurological severity score (mNSS) were pronounced after TBI and decreased (*p* < 0.05) with MEL. KCC2 expression was downregulated after TBI and increased with MEL. Expression of BDNF and p-ERK was down-regulated by TBI and attenuated by MEL (*p* < 0.05). Apoptosis increased after TBI and was reduced with MEL (*p* < 0.05).
**Conclusion**	Many signaling changes occur after TBI which contribute to diverse histological and functional deficits. MEL shows promise for targeting TBI outcomes. Additional studies are needed especially those evaluating the longer-term effects of MEL.
